# The role of a community conversation intervention in reducing stigma related to lower limb lymphoedema in Northern Ethiopia

**DOI:** 10.1186/s12913-024-10864-w

**Published:** 2024-03-19

**Authors:** Abebayehu Tora, Stephen Bremner, Oumer Ali, Mersha Kinfe, Asrat Mengiste, Vasso Anagnostopoulou, Abebaw Fekadu, Gail Davey, Maya Semrau

**Affiliations:** 1 Department of Sociology, Wolaita Sodo University, Wolaita Sodo, Ethiopia; 2 Center for Innovative Drug Development and Therapeutic Trials for Africa (CDT-Africa), Addis Ababa University, Addis Ababa, Ethiopia; 3 Centre for Global Health Research, Brighton and Sussex Medical School (BSMS), Brighton, UK; 4 Armauer Hansen Research Institute, Addis Ababa, Ethiopia; 5 School of Public Health, Addis Ababa University, Addis Ababa, Ethiopia

**Keywords:** Lower-limb lymphoedema, Stigma, Community conversation

## Abstract

**Background:**

Stigma related to lower-limb lymphoedema poses a major psychosocial burden to affected persons and acts as a barrier to them accessing morbidity management and disability prevention (MMDP) services. Community Conversation (CC), which actively engages community members and disseminates health information amongst them, is believed to break the vicious cycle of stigma by enhancing disease-related health literacy at the community level.

**Methods:**

A quasi-experimental study was conducted in Northern Ethiopia to assess the role of the CC intervention in reducing stigma. In two control districts, a comprehensive and holistic MMDP care package was implemented that included physical health, mental health and psychosocial interventions, whilst in the intervention district the CC intervention was added to the MMDP care package. A total of 289 persons affected by lymphoedema and 1659 community members without lymphoedema were included in the study.

**Results:**

Over the course of the intervention, in all sites, community members’ knowledge about the causes of lymphoedema increased, and perceived social distance and stigmatizing attitudes towards people with lymphoedema decreased in the community, whilst experienced and internalized stigma decreased amongst affected persons. There were no significant changes for perceived social support. However, the changes were greater in the control sites overall, i.e. those districts in which the holistic care package was implemented without CC.

**Conclusion:**

The findings suggest that the CC intervention provides no additional stigma reduction when used alongside a holistic MMDP care package. Provision of comprehensive and holistic MMDP services may be adequate and appropriate to tackle stigma related to lower-limb lymphoedema in a resource-constrained setting like Ethiopia.

**Supplementary Information:**

The online version contains supplementary material available at 10.1186/s12913-024-10864-w.

## Background

Neglected tropical diseases (NTDs) that result in lower-limb lymphoedema (i.e. swelling of the lower leg) include lymphatic filariasis (LF), leprosy and podoconiosis. Global burden estimates for people affected by LF lymphoedema, podoconiosis and leprosy are 15 million, 4 million and 2 to 3 million respectively [1, 2]. Nationwide mapping in 2013 demonstrated that podoconiosis accounts for approximately 64.8%, LF for 13.2%, and leprosy for 12.8% of the total burden of lymphoedema in Ethiopia [3].

Stigma related to lower-limb lymphoedema is one of the key issues that significantly increases its disease burden [4] and acts as a major barrier to accessing morbidity management and disability prevention (MMDP) services [5, 6]. The prejudice, discrimination and internalized stigma related to lower-limb lymphoedema not only compromise the psychological and social wellbeing of affected persons and their families but can also limit their access to healthcare and adherence to treatment [5]. This can lead to a vicious cycle, creating further disability, illness, and reduced economic productivity [7]. The three major sources of stigma for lower-limb lymphoedema are: i) misinformation about its causes, treatment and prevention amongst the community, affected persons and their families; ii) the associated poverty and reduced quality of life due to affected individuals’ lost economic productivity; and iii) the economic burden related to the costs of care, including transport to health facilities [8–11]. People affected by podoconiosis, leprosy and LF are stigmatized in all areas of their daily life. Social functions such as partner selection and marriage, employment, and participation in community leadership are common domains of life in which affected persons and their families experience discrimination. These deep-seated stigmatizing experiences can cause internalized stigma in affected persons, i.e. acceptance of stigmatizing social views and resultant feelings of shame, guilt or fear, as a result of the experience of discrimination. Internalized stigma is manifested in the form of low self-esteem, suicidal ideation, and avoidance of interactions with non-affected community members. [12]. Breaking the vicious cycle of stigma related to lymphoedema has therefore become a top priority for various community organizations and researchers. Strategies to address this stigma have included: i) educational interventions; ii) community-based socio-economic rehabilitation; and iii) providing integrated services in nearby health facilities at low or no cost [13]. Interventions often aim to remove the drivers of stigma and address the norms and policies that facilitate the stigmatization process. The drivers of stigma include fear of infection through contact, concerns about productivity due to poor health, social judgment and blame. Facilitators of stigma include social isolation and lack of social support [8, 14, 15].

Studies have suggested that multi-component interventions are more effective than single-component interventions for stigma reduction [16, 17]. Multi-component stigma reduction interventions target both the stigmatizers and the stigmatized to ensure holistic and sustainable changes in outcomes. These interventions support individuals with health-related stigma to cope with experienced stigma (i.e. actual experiences of discrimination) and overcome internalized stigma, as well as reaching out to community members to shift harmful notions about health conditions through community dialogues or engaging local leaders to share anti-stigma messages [18–21].

Implementing and donor actors now widely recognize that joint approaches to reduce stigma across NTDs may be feasible given the similarities in causes, manifestations and interventions [22], but there remains a knowledge gap in regard to relevant, evidence-based stigma reduction interventions for use within integrated MMDP programmes. To address this knowledge gap, an implementation research project titled ‘Improving access to integrated Morbidity management and disability PREvention Services through Stigma reduction for people with lower limb lymphoedema in Ethiopia’ (IMPRESS) was implemented in selected districts of Northern Ethiopia where LF, podoconiosis and leprosy-related lymphoedema are prevalent. The IMPRESS project was embedded within the ‘Excellence in Disability Prevention Integrated across NTDs’ (EnDPoINT) programme, which ran from 2017 to 2021 in response to a request by the Federal Ministry of Health (FMOH) in Ethiopia. Details in regard to the development and implementation of the EnDPoINT/IMPRESS project have been provided in previous reports [23]. The EnDPoINT care package entailed comprehensive MMDP and mental health services including capacity-building training for health care organization staff, case detection and treatment for patients at health facility level, integrated mental health care, and community awareness campaigns [23–26]. The IMPRESS study built on this unique opportunity and focused on the specific question of how best to reduce stigma within this integrated MMDP programme. EnDPoINT/IMPRESS was guided by the World Health Organization’s Community-Based Rehabilitation (CBR) strategy [27], as it promotes inclusion and participation of marginalized groups through multi-sectoral interventions across five key domains (health, education, livelihood, social, and empowerment). The CBR model acknowledges that programmes need to go beyond the health domain to empower affected persons to take an active role in their development, as it supports equity in services by building capacity amongst affected persons and their communities [28]. The IMPRESS study embedded Community Conversation (CC) as one of several intervention components within EnDPoINT’s holistic care package [23] with the aim to increase disease-related health literacy at the community level, reduce stigma and improve access to MMDP services. In other disease contexts including HIV and mental health, studies have documented the positive role of CC in stigma reduction [29]. In our previous qualitative process evaluation report from IMPRESS [30], the acceptability and feasibility of integrating CC into the lymphoedema care package and routine primary health care services was documented. This report also highlighted the potential role of CC in raising awareness and overcoming stigmatizing attitudes within the community. However, the role of CC in reducing stigma when added to the holistic MMDP care package had not been explored quantitatively. The study presented here therefore aimed to quantitatively investigate whether CC contributed significantly to stigma reduction when added into EnDPoINT’s integrated holistic care package.

## Methods

### Study setting

This study was conducted in Awi Zone, one of the ten zones in Amhara regional state of Ethiopia. The zone is located 469 km north of Addis Ababa, the capital city of Ethiopia. The zone is divided into three urban and nine rural districts and covers a geographic area of 9,148 square kilometers. The elevation varies from 1,800 to 3,100 m above sea level, with an average altitude of about 2,300 m. Awi zone was selected as the study site because of the established co-endemicity of podoconiosis, LF and leprosy, and because it represented the climatic diversity found within Ethiopia. Awigna and Amharic are the main languages spoken in Awi zone.

### Community conversation intervention

Details about the development and implementation of the CC intervention are available in our previous process evaluation report [30]. A total of 33 CC facilitators were recruited from the intervention district who participated in three days training using a standardized CC facilitation guide. The CC facilitation guide described the principles and procedures of health communication and outlined health messages about the causes, treatment and prevention of lower limb lymphoedema. Three CC facilitators were deployed in each *kebele* (lowest level administrative unit) to form a CC group of 30–50 participants constituting both affected and unaffected community members. A total of 400 participants engaged in the first CC session across 11 groups. Each CC group was expected to participate in two CC sessions per month. Each CC group member was expected to attend a maximum of six sessions over a three-month period. CC participants were required to disseminate health information to at least five community members per month through gatherings for various social occasions in their locality.

### Study design

**Fig. 1:**
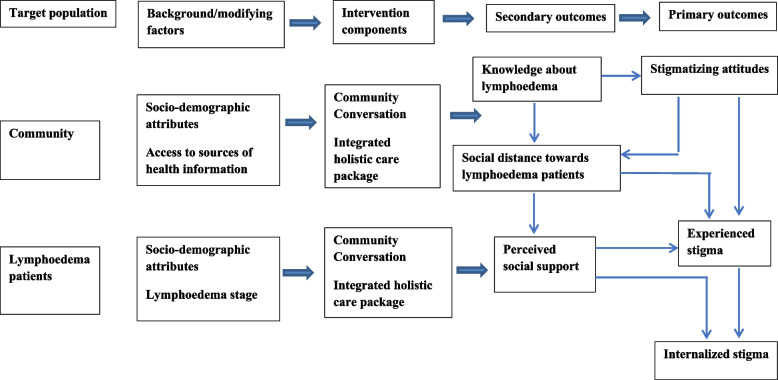
Conceptual framework indicating the intervention effects of community conversation on study outcomes

A quasi-experimental design was employed to evaluate the role of CC in reducing stigma related to lower-limb lymphoedema. Of the three EnDPoINT scale-up districts in Awi zone (Ankisha, Guangua and Guagusa Shikugiad) [23], CC was implemented in Guagusa Shikugiad district (the intervention district) for six months as an addition to the EnDPoINT integrated MMDP holistic care package. CC was delayed in the other two districts (Ankisha and Guangua districts) for control purposes, though the other components of the EnDPoINT care package continued to take place. The CC intervention was implemented over a period of six months, with outcomes being assessed at baseline and at six-month follow-up (endline). Figure 1 presents a conceptual framework that includes the patient and community outcome variables used in this study and the pattern of relationships between these variables. Patient outcome variables included experienced stigma, internalized stigma, and perceived social support. Community outcome variables included knowledge about the causes of lymphoedema, stigmatizing attitudes towards persons affected by lymphoedema, and social distance towards affected persons. Exposure of affected persons and community members to CC was intended to bring about attitudinal and behavioural changes in both groups. Socio-demographic factors such as age, gender, education, occupation, income, lymphoedema stage (for the patient cohort) and access to health information (for the community cohort) were included in the conceptual framework as intervening variables. Community members’ access to sources of health information was assessed using questions about frequency of exposure to sources such as magazines, radio, television, health education sessions and meetings.

### Study outcome measures

#### Community member outcomes

##### Knowledge about lymphoedema

Community members’ knowledge about the causes of lymphoedema was assessed using a purposively developed questionnaire that included a total of fourteen indicators that were identified from the podoconiosis, LF and leprosy literature [8, 9]. Responses were scored 0, indicating ‘Yes’, or 1 indicating ‘No’. Higher scores represented better knowledge about the causes of lymphoedema. The minimum and maximum total scores for the index were 0 and 14 respectively.

##### Attitudes towards persons affected by lymphoedema

Attitudes of the community towards persons affected by lymphoedema were assessed using an index constituting 13 negatively framed statements that were identified from previous reports [8–10]. Possible responses were 0 for ‘No’ and 1 for ‘Yes’, where lower scores indicated more positive attitudes. The minimum and maximum total scores of the attitude index were 0 and 13 respectively.

##### Social distance

The degree of closeness of community members towards persons affected by lymphoedema was assessed using the 7-item Social Distance Scale (SDS) [19, 31], which was adapted to the study context. The response scale ranged from 1 (‘none of the time’) to 5 (‘all of the time’) for 6 of the questions, and 0 to 3 for one of the questions. The minimum and maximum total scores for the scale were 6 and 33, where lower scores indicated higher willingness to have social interactions or contacts with persons affected by lymphoedema.

#### Patient outcomes

##### Experienced stigma

To measure experienced stigma amongst affected persons, the discrimination section of the DISC-12 scale [32] was used. The 22-item discrimination section of the original DISC-12 was modified as described in a previous Ethiopian study, by removing two items not applicable to the rural Ethiopian setting of the study and two items with low item-factor loading [33]. Thus, in this study, 18 items were included with a response scale rating of 0 to 3. The possible total scores ranged from 0 to 54, with higher scores reflecting more discrimination.

##### Internalized stigma

Indicators for internalized stigma were adapted from the Internalized Stigma in Mental Illness (ISMI) scale [34]. The 29-item ISMI scale was originally developed for mental illness and was adapted by experts in lymphoedema to produce an 11-item score relevant to lymphoedema, with a response scale rating of 1 to 4. The range of scores possible in this study was 11 (less internalized stigma) to 44 (more internalized stigma).

##### Perceived social support

Perceived social support was measured using the Oslo Social Support tool (Oslo-3) [35], a brief 3-item scale that assesses the number of close confidantes, perceived level of concern from others, and perceived ease of getting help from neighbors. The response scale ratings ranged from 1 to either 4 or 5 (for different questions). The total score ranges from 3 to 13 and higher scores represent better social support. The Oslo-3 scale has been used previously in Ethiopia in a study that examined the prevalence of, and factors associated with, depression among patients with HIV/acquired immunodeficiency syndrome (AIDS) [36], as well as a study addressing the prevalence and predictors of depression among pregnant women [37].

#### Sampling and sample size

The study populations were persons affected by lower-limb lymphoedema caused by podoconiosis, LF or leprosy, and unaffected community members. In the community sample, to detect a small effect size of 0.2 (Cohen’s D) for the difference between intervention and control responses, assuming a correlation between baseline and endline measurements of 0.6 [38], with twice as many respondents from control sites as at the intervention site, with 90% power for 5% significance, required a sample size of 759 respondents at both baseline and endline. We anticipated many respondents not to be at home when the endline visit took place; allowing for 35% attrition at endline required 1,168 community respondents at baseline.

Affected persons were those identified as requiring care for their lymphoedema and/or comorbid mental ill-health. They were identified and recruited into the study based on their case identification within the EnDPoINT programme within which this study was embedded. Adults (≥ 18 years of age) with lower-limb lymphoedema caused by LF, podoconiosis and/or leprosy were identified and invited into the study by community health extension workers during routine home visits in the three study districts (i.e. the one CC intervention district and the two control districts). To detect a medium-small effect size of 0.35 (Cohen’s D) for the difference between intervention and control responses, assuming a correlation between baseline and endline measurements of 0.6, with twice as many respondents from control sites as at the intervention site, with 90% power for 5% significance, required a sample size of 249 at both baseline and endline. Allowing for 10% attrition at endline required 277 patient respondents at baseline.

#### Data collection and management

Both the patient and community survey questionnaires were originally prepared in English and were then translated into the local language, Amharic. Consistency of meaning in translation and back-translation was checked by the research team. The questionnaires were field-tested to determine understandability of the questions. Experienced data collectors were recruited and trained in how to use the study tools and how to extract information from both written and electronic health records. Regular supervision of the data collection process was carried out by the investigating team. All completed questionnaires and tools were checked for consistency, completeness, clarity and accuracy. Data were collected by smartphones using Open DataKit, free software developed to allow data collection using Android mobile devices and data submission to an online server. After collection, data were transferred to an Excel (Microsoft, Redmond, WA, USA) datasheet for cleaning and verification, before being exported to Stata version 17 (StataCorp. 2015. *Stata Statistical Software: Release 14*. College Station, TX: StataCorp LP.) for analysis.

#### Data analysis

Community and patient cohort data are described separately, the outcomes being compared by time point and whether participants resided in the CC intervention site or a control site. Summary statistics appropriate to distribution are presented, i.e. mean and standard deviation for normally distributed variables, median and interquartile range for skewed continuous variables, and frequency and percentage for categorical variables. To use all available data at each time point, linear mixed effects models were fitted to the ‘Stigmatizing Attitudes’, ‘Social Distance Scale’ and ‘Knowledge about lymphoedema’ outcomes for the community cohort, and the ‘Experienced stigma’, ‘Internalized Stigma’ and ‘Social Support’ outcomes for the patient cohort. The basic model for each outcome was specified as follows: observations at baseline (T0) and 6-month follow-up (T1) were modelled as repeated measures so that change from baseline could be estimated as a fixed effect, as well as the difference in average outcome scores between CC and control participants and (where statistically significant) the interaction between treatment and time (i.e. CC vs. control × T1 vs T0). A random effect was included for participants to allow for the repeated measurements. This approach means that participants present only at one of the two time points still contributed information to the models. In the community cohort, we adjusted each model for the following baseline characteristics: age, gender, educational attainment, marital status, perceived economic status, occupation and ‘access to health information’ score. In the patient cohort, we adjusted for age, gender, educational attainment, marital status, occupation, relative income and lymphoedema stage (as a continuous variable). For each of these six models, we removed variables that were not statistically significant and present the final parsimonious models. For each of the effects we present the regression coefficient, its 95% confidence interval and p-value. In addition, we present the model-based intra-cluster correlation coefficient (ICC) and its 95% confidence interval (CI). Further, sensitivity analyses were conducted for the community cohort to fit models including only participants who were sampled at both time points.

## Results

### Socio-demographic characteristics of community cohort

The number of community respondents at baseline was 1211 in the three districts in total – 385 (31.8%) in the CC intervention district and 826 (68.2%) in the control districts. Table 1 presents the baseline summary statistics of the socio-demographic characteristics of community respondents. The average age, sex and literacy of participating community members were very similar at the intervention and control sites. Participants were on average 40 years of age, 60% were male, and over 30% were illiterate, with less than 10% attending any tertiary education. Around three-quarters were married, and farming was the dominant occupation for respondents at both the intervention and control sites. Just under half of all respondents perceived their economic status to be average and reported relatively good access to health information sources with mean scores of 12.5 and 13.0 (out of a possible 15) respectively.

**Table 1:** Socio-demographic characteristics of community respondents at baseline

**Variable**	**Category**	**Intervention site**		**Control sites**		**Total**	
		n	%/mean (SD)	n	%/mean (SD)	N	%/mean (SD)
**Age (Years)**	-	385	39.8 (14.9)	826	40.6 (14.7)	1211	40.3 (14.7)
**Sex**	Male	241	62.6	473	57.3	714	59.0
	Female	144	37.4	353	42.7	497	41.0
	Total	385	100.0	826	100.0	1211	100.0
**Education**	Cannot read or write	118	30.6	277	33.5	395	32.6
	No formal schooling but can read or write	89	23.1	254	30.8	343	28.3
	Formal schooling (grades 1–12)	142	36.9	257	31.1	399	32.9
	University/college diploma	36	9.4	38	4.6	74	6.1
	Total	385	100.0	826	100.0	1211	100.0
**Marital Status**	Single	67	17.4	124	15.0	191	15.8
	Married	279	72.5	620	75.1	899	74.2
	Separated/divorced	17	4.4	49	5.9	66	5.5
	Widowed	22	5.7	33	4.0	55	4.5
	Total	385	100.0	826	100.0	1211	100.0
**Occupation**	Housewife	34	8.8	65	7.9	99	8.2
	Farmer	205	53.2	610	73.8	815	67.3
	Merchant/ Petty trader	40	10.4	24	2.9	64	5.3
	Student	44	11.4	88	10.7	132	10.9
	Civil servant	39	10.1	7	0.8	46	3.8
	Daily laborer	11	2.9	16	1.9	27	2.2
	Other	12	3.1	16	1.9	36	3.0
	Total	385	100.0	826	100.0	1211	100.0
**Economic status**	Poor	146	37.9	304	36.8	450	37.2
	Average	161	41.8	337	40.8	498	41.1
	Better off	78	20.3	185	22.4	263	21.7
	Total	385	100.0	826	100.0	1211	100.0
**Access to sources of health information (Index scores)**	-	385	12.5 (1.9)	826	13.0 (1.8)	1211	12.9 (1.8)

### Socio-demographic characteristics of patient cohort

The total number of patient cohort respondents was 289 at baseline. Of these, 68 respondents were from the intervention site and 221 from control sites. Table 2 describes the baseline summary statistics of the socio-demographic characteristics of patient cohort respondents. The socio-demographic profile of patient cohort survey participants was similar in both intervention and control sites. Participants were on average 50 years of age, and over half were female. Nearly two thirds of participants were married and a similar proportion were illiterate. Farming was the dominant occupation for over 78% of participants. Over half of respondents perceived their income to be low, whilst 28.7% perceived it to be middle-ranking. In terms of lymphoedema condition, over 57% of participants were at stage 3.

**Table 2:** Socio-demographic characteristics of patient respondents at baseline

Variables	Category	Intervention site		Control sites		Total	
		n	% / mean (sd)	n	% / mean (sd)	n	% / mean (sd)
**Age (years)**	-	68	50.6 (14.5)	221	50.6 (13.0)	289	50.6 (13.3)
**Sex**	Male	30	44.1	108	48.9	138	47.8
	Female	38	55.9	113	51.1	151	52.2
	Total	68	100	221	100	289	100
**Education**	Cannot read or write	41	60.3	142	64.3	183	63.3
	No formal schooling but can read and write	23	33.8	63	28.5	86	29.8
	Attended formal education	4	5.9	16	7.2	20	6.9
	Total	68	100	221	100	289	100
**Marital status**	Single	4	5.9	6	2.7	10	3.5
	Married	39	57.4	143	64.7	182	63
	Separated/divorced	21	30.9	38	17.2	59	20.4
	Widowed	4	5.9	34	15.4	38	13.1
	Total	68	100	221	100	289	100
**Occupation**	Paid work	0	0	1	0.5	1	0.3
	Own business	5	7.4	15	6.8	20	6.9
	Farming	54	79.4	173	78.3	227	78.5
	Housewife	6	8.8	28	12.7	34	11.8
	Student	1	1.5	2	0.9	3	1
	Unemployed	0	0	1	0.5	1	0.3
	Other	2	2.9	1	0.5	3	1
	Total	68	100	221	100	289	100
**Relative income**	Very low	11	16.2	29	13.1	40	13.8
	Low	39	57.4	122	55.2	161	55.7
	Middle	17	25	66	29.9	83	28.7
	High	1	1.5	2	0.9	3	1
	Very high	0	0	2	0.9	2	0.7
	Total	68	100	221	100	289	100
**Lymphoedema stage left**	Stage 0	2	2.9	5	2.3	7	2.4
	Stage 1	6	8.8	11	5	17	5.9
	Stage 2	14	20.6	62	28.1	76	26.3
	Stage 3	36	52.9	130	58.8	166	57.4
	Stage 4	6	8.8	8	3.6	14	4.8
	Stage 5	4	5.9	5	2.3	9	3.1
	Total	68	100	221	100	289	100
**Lymphoedema stage right**	Stage 0	2	2.9	3	14	5	1.7
	Stage 1	5	7.4	13	5.9	18	6.2
	Stage 2	14	20.6	63	28.5	77	26.6
	Stage 3	40	58.8	127	57.5	167	57.8
	Stage 4	4	5.9	10	4.5	14	4.8
	Stage 5	3	4.4	5	2.3	8	2.8
	Total	68	100	221	100	289	100

### Community survey outcomes

Out of 1211 respondents participating in the baseline survey, 759 were present at both baseline and endline, while 452 respondents participated only at baseline. Additionally, 448 newly recruited respondents participated in the endline survey giving a total sample of 1659. Table 3 presents summary statistics for the paired baseline and follow-up scores of community cohort outcomes, namely knowledge, stigmatizing attitudes and perceived social distance in the intervention and control sites. The baseline mean (SD) scores for community knowledge about causes of lymphoedema among respondents in the intervention and control sites were 7.2 (1.7) and 7.2 (1.9) respectively. At follow-up, an increase in mean knowledge scores was observed in both the intervention and control sites, to 7.8 (1.7) and 8.0 (1.6) respectively. At baseline, the mean scores for stigmatizing attitudes of community respondents in the intervention and control sites were 3.3 (1.8) and 2.9 (1.8) respectively. A reduction in the mean scores for stigmatizing attitudes of community respondents was observed over time to 2.3 (1.6) and 2.1 (1.5) in the intervention and control sites respectively. The baseline mean scores for perceived social distance among community respondents were similar in the intervention and control sites, 17.9 (6.6) and 18.1 (6.0) respectively. A reduction in mean scores was observed over time to a mean score of 17.1 (5.2) in the intervention site and 16.8 (5.0) in the control sites.

**Table 3:** Knowledge, stigmatizing attitudes and perceived social distance among community respondents present at both time points

Present both time points	Direction	Time point	Intervention site			Control sites			Total		
			n	mean	sd	n	mean	sd	n	mean	sd
**Knowledge about causes of lymphoedema**	Higher better	Baseline	218	7.2	1.7	541	7.2	1.9	759	7.2	1.8
		Follow-up	218	7.8	1.7	541	8.0	1.6	759	8.0	1.6
**Stigmatizing attitudes**	Lower better	Baseline	218	3.3	1.8	541	2.9	1.8	759	2.9	1.8
		Follow-up	218	2.3	1.6	541	2.1	1.5	759	2.1	1.6
**Social distance**	Lower better	Baseline	218	17.9	6.6	541	18.1	6.0	759	18.1	6.2
		Follow-up	218	17.1	5.2	541	16.8	5.0	759	16.9	5.1
**Present baseline only**											
** Knowledge about causes of lymphoedema**	Higher better	Baseline	167	7.2	1.7	285	7.2	1.7	452	7.4	1.7
** Stigmatizing attitudes**	Lower better	Baseline	167	3.2	1.8	285	3.2	1.9	452	3.2	1.9
** Social distance**	Lower better	Baseline	167	17.2	6.8	285	18.8	5.9	452	18.2	5.9
**Present endline only**											
** Knowledge about causes of lymphoedema**	Higher better	endline	169	7.7	1.8	279	8.0	1.6	448	7.9	1.7
** Stigmatizing attitudes**	Lower better	endline	169	2.2	1.6	279	2.5	1.6	448	2.4	1.6
** Social distance**	Lower better	endline	169	17.0	5.1	279	16.9	5.2	448	16.9	5.2

### Patient cohort outcomes

The baseline and follow-up mean scores of patient cohort outcomes (perceived social support, experienced stigma and internalized stigma) are presented in Table 4. The baseline mean scores for perceived social support in the intervention and control sites were 6.6 (2.3) and 7.1 (2.6) respectively. The observed change in mean scores at follow-up compared to baseline was very small; the follow-up mean scores were 6.8 (2.4) and 7.0 (2.4) in the intervention and control sites respectively. The median (IQR) scores for experienced stigma at baseline were 0.63 (0.72) in the intervention site and 0.52 (0.46) in the control sites. A reduction in median score for experienced stigma was observed at follow-up, to 0.38 (0.44) and 0.28 (0.24) in the intervention and control sites respectively. There was also a reduction in mean scores observed for internalized stigma between baseline and follow-up, from 28.0 (5.3) at baseline to 24.9 (5.9) at follow-up in the intervention site, and from 26.7 (5.8) to 22.8 (4.8) in the control sites.

**Table 4:** Perceived social support, experienced stigma and internalized stigma among patient cohorts at each time point

Variables	Direction	Time point	Intervention site			Control sites			Total		
			n	Mean/ median	SD/IQR	n	Mean/median	SD/IQR	n	Mean/median	SD/IQR
**Perceived social support**	Higher better	Baseline	68	6.6	2.3	221	7.1	2.6	289	7.0	2.5
		Follow-up	63	6.8	2.4	192	7.0	2.4	255	6.9	2.4
**Experienced stigma** ^a^	Lower better	Baseline	68	0.63	0.72	221	0.52	0.46	289	0.54	0.51
		Follow-up	68	0.38	0.44	192	0.28	0.24	255	0.30	0.26
**Internalized stigma**	Lower better	Baseline	68	28.0	5.3	221	26.7	5.8	289	27.0	5.7
		Follow-up	63	24.9	5.9	192	22.8	4.8	255	23.4	5.2

^a^Summary statistics are median and interquartile range (IQR)

### Intervention and time effects from adjusted models for community and patient cohort outcomes

#### Modelling results

A total of 1617 community respondents were included in the models. The total sample at baseline was 1211 but a further 448 were present at endline who were not present at baseline, so the total sample size was 1659. However, the models are fitted to 1617 observations due to missing data. The model results presented in Table 5 are based on all of the data. Sensitivity analysis was conducted whereby only participants who were present at both time points of the community cohort were included. The conclusions do not change. Apart from time and intervention effects, other variables not significant at the 5% level were removed from the models to create simpler (more parsimonious) models. In all models, except internalized stigma (patient cohort), the intra-cluster correlation coefficients (ICCs) were large. Table 5 shows the treatment (CC vs. control), time (T1 vs. T0) and treatment-by-time interaction effects (where significant) for each outcome. The confounding factors that remained significant in the models are listed in the footnote but the estimates for these are not shown here.

**Table 5:** Intervention and time effects from adjusted models for community survey and patient cohort outcomes

Community survey (*N* = 1617)	Comparison	Coefficient	95% C.I	*p*-value	Model ICC	95% C.I
**Stigmatizing attitudes** ^a^	CC vs. Control	0.407	(0.187, 0.627)	< 0.001	0.26	(0.18, 0.34)
	T1 vs T0	-0.700	(-0.848, -0.553)	< 0.001		
	Treat × Time	-0.322	(-0.599, -0.046)	0.022		
**Social distance** ^b^	CC vs. Control	-0.187	(-0.979, 0.605)	0.644	0.33	(0.27, 0.39)
	T1 vs T0	-1.442	(-1.890, -0.993)	< 0.001		
	Treat × Time	0.944	(0.049, 1.840)	0.039		
**Knowledge** ^a^	CC vs. Control	0.058	(-0.165, 0.281)	0.608	0.28	(0.22, 0.35)
	T1 vs T0	0.850	(0.701, 0.998)	< 0.001		
	Treat × Time	-0.438	(-0.703, -0.172)	0.001		
**Patient cohort (** ***N*** ** = 289)**						
** Social support** ^d^	CC vs. Control	-0.290	(-0.782, 0.202)	0.248	0.19	(0.09, 0.35
	T1 vs T0	-0.061	(-0.428, 0.306)	0.744		
** Experienced stigma** ^c^	CC vs. Control	0.110	(0.027, 0.193)	0.009	0.11	(0.03, 0.36)
	T1 vs T0	-0.241	(-0.297, -0.186)	< 0.001		
** Internalized stigma** ^c^	CC vs. Control	1.764	(0.717, 2.812)	0.001	< 0.001	
	T1 vs T0	-3.650	(-4.544, -0.276)	< 0.001		

^a^Adjusted for occupation and educational attainment

^b^Adjusted for access to health information score, sex and occupation

^c^Adjusted for age and educational attainment

^d^Adjusted for educational attainment and relative income

There was no evidence of a difference in knowledge of the causes of lymphoedema between the CC intervention and control groups. The score was higher at follow-up than at baseline by 0.85 points (95% CI 0.70, 1.00; *p* < 0.001). There was strong evidence of a difference in effects over time in CC participants versus control participants. The mean stigmatizing attitudes score was 0.41 points higher in CC participants than control participants (95% CI 0.19, 0.63; *p* < 0.001). There was strong evidence that this score was lower at follow-up than at baseline, a mean decrease of -0.70 points and that the change over time was different between the CC and control groups (95% CI -0.85, -0.55; *p* < 0.001). There was no evidence of a difference in social distance between CC and control participants, though there was a change in mean score over time of -1.44 points (95% CI -1.89, -0.99; *p* < 0.001).

There was a total of 289 participants in the patient cohort. The experienced stigma score was slightly greater on average in CC participants than in controls, by 0.11 points (95% CI 0.03, 0.19; *p* < 0.01) and was lower at follow-up than at baseline by -0.24 points (95% CI -0.30, -0.19; *p* < 0.001). The internalized stigma score was greater in CC participants than in controls by an average of 1.76 points (95% CI 0.72, 2.81; *p *< 0.01) but was significantly lower at follow-up than at baseline by an average of -3.6 points (95% CI -4.54, -0.28; *p* < 0.001). There was no evidence of a difference in perceived social support between CC or control participants nor of a change over time.

## Discussion

The stigma associated with the three skin NTDs studied is known to affect the psychosocial wellbeing of affected persons and their associates and obstructs access to health care services. Empirical evidence from other health conditions supports the importance of CC in reducing stigma through enabling participants to set a plan of action, develop a sense of common purpose, overcome fear, denial and passivity and move from being passive recipients of health information to active problem solvers [29]. CC has been shown to be vital for enabling healthy behaviours, facilitating timely and appropriate access to health services and supporting optimum treatment adherence [39, 40]. Some community-based organizations (CBOs) in Ethiopia have applied a CC approach to tackle stigma and promote access to lymphoedema care services [41, 42], though quality of the implementation process and outcomes of these efforts have not been adequately documented. Though community-based organizations have also been implementing CC as a vertical program, little is known about the feasibility of integrating CC into the primary health care system in combination with MMDP services. Embedded within the EnDPoINT programme [24], the IMPRESS project – to our knowledge – made the first effort of its kind to integrate CC into the primary health care system to tackle stigma related to lower-limb lymphoedema.

This study revealed findings that are relevant for decision makers, implementers and researchers. The intervention effect of CC was mixed. On the one hand, no difference was observed between the intervention and control districts in terms of improvements in knowledge about the causes of lymphoedema, stigmatizing attitudes and stigmatizing practice (perceived social distance and social support). These are the key drivers of social stigma in lymphoedema patients [8, 9]. On the other hand, the time effects were significant in both the intervention and control districts. Changes were observed over a six-month period of CC implementation: community members’ knowledge about the cause(s) of lymphoedema improved, and stigmatizing attitudes and perceived social distance reduced. These changes may be accounted for directly or indirectly by the EnDPoINT holistic care package. The EnDPoINT pilot study (without CC) suggested an association between the holistic care package and improved quality of life, reduced levels of depression, and reduced experiences of internalized stigma and discrimination [26]. A range of studies have also demonstrated that encouraging simple self-care measures to promote foot hygiene can reduce limb swelling and improve quality of life [43–45].

Positive changes (reductions in stigmatizing attitudes, social distance, experienced and internalized stigma) were observed in intervention and control districts over time. Unexpectedly, these changes were larger in the control districts. This might be explained by low quality of implementation of CC in the intervention district. Our previous qualitative process evaluation [30] documented a range of challenges affecting quality of CC implementation. Some of these challenges were: inadequacy of training and supportive supervision, perceived complexity of the CC facilitation guide, lack of competence to run CC sessions according to the principles and procedures in the guide, and low motivation and commitment of CC facilitators to run the CC sessions as planned due to absence of incentives. These challenges are likely to have compromised the quality of outcomes of the CC intervention, resulting in misunderstanding of health messages about lymphoedema. A study that applied a Lay Health Advisor model in southern Ethiopia also observed misunderstanding of health messages due to the low competence of Lay Health Advisors in communicating complex health messages to recipients [46]. The smaller reduction in internalized and experienced stigma in CC intervention districts may also be associated with low competence of CC facilitators.

As this study reports changes observed over six-month follow-up, longer-term follow-up assessments may better capture the association between the implementation of the CC intervention and its outcomes. Hence, for broader understanding of the role of an integrated CC intervention as a stigma reduction strategy in the context of skin-NTDs, future research may consider longer-term follow-ups to assess the sustained impact of the quality of implementation of the CC intervention on stigma and community attitudes towards lower-limb lymphoedema. Additionally, though patients and community members participated actively in the design and implementation of the interventions, inadequate feedback mechanisms for ensuring their satisfaction with the process and outcomes of their engagement may be a limitation of this study.

An economic evaluation of the EnDPoINT care package is currently underway. This suggests that effectiveness of the EnDPoINT care package combined with the CC intervention may be slightly higher than usual care (i.e. the EnDPoINT care package alone), but that CC is unlikely to be good value for money given the high values of incremental cost-effectiveness ratio (ICER), i.e. cost per one unit change in effectiveness outcome (N. Ivashikina, Personal Communication). Coupled with the implementation challenges reported previously [30], the high economic cost of integrating CC into the primary health care system is likely to be an additional burden in resource-constrained settings.

As this study addressed only the added value of CC to the EnDPoINT care package in reducing stigma related to lower limb lymphoedema, our findings should be interpreted only in the context of MMDP services integrated into primary health facilities for lymphoedema patients, rather than as standalone intervention. The unexpected observations, particularly in control districts, do not nullify the documented positive role of standalone CC interventions implemented in community settings, for example in competence levels in HIV [29, 39, 40].

In conclusion, adding CC to a holistic care package integrated into the primary health care system may not result in any further gains in tackling stigma related to lymphoedema than those already brought about by the care package. Hence, policy-makers and practitioners may prioritize integration of the EnDPoINT care package into the primary health care system instead of the CC intervention for efficient utilization of financial and human resources.

### Supplementary Information

**Additional file 1.** Community survey questionnaire.

**Additional file 2.** Patient cohort survey questionnaire.

## Data Availability

All data generated or analysed during this study are included in this published article.
