# Supplementary material for: The role of a community conversation intervention in reducing stigma related to lower limb lymphoedema in Northern Ethiopia
Source: BMC Health Serv Res. 2024 Mar 19;24:353. doi: 10.1186/s12913-024-10864-w (PMC10949692; doi:10.1186/s12913-024-10864-w)
Supplement: Supplementary file 1 — Additional file 1. Community survey questionnaire. [file 12913_2024_10864_MOESM1_ESM.pdf]

Lymphoedema in Awi zone
Community Survey questionnaire

Identification

**Woreda:**	**Kebele**:	**Gott** or **Cluster No (Enumeration are):**

Time interview started:__________________: Time interview ended: ______________

DATA COLLECTOR:
Name:_________________________
Date:__________________________
Signature: _____________________

SUPERVISOR:
Name:___________________________
Date:____________________________
Signature: _______________________

NO	QUESTIONS AND FILTERS	CODING CATEGORIES	SKIP	Ansr
**Section 1: Background**				
101	Name of respondents			
102	How old are you? (**in completed years**)	................Years		
103	What is your religion?	Orthodox…1 Protestant …. 2 Muslim … 3<br>Other 88		
104	What is your ethnicity?	Amhara……1 Oromo…….2<br>Other (Specify)… 88		
105	What is your occupation, that is, what kind of work do you mainly do as a means of livelihood?	Housewife……….......... 1 Farmer…..... 2<br>Merchant/ Petty trader …3 Student ....4<br>Civil servant…………… .5 Daily laborer…..6<br>Other 88		
106	What is the highest grade you completed?	Cannot read and write …….0 No formal school but can read & Write. .... 2<br>University/College diploma… .... 3<br>Formal education grade……..		
107	Are you currently attending formal school?	Yes …………1 No.............0		
108	What is your marital status	Single……1<br>Married… .. 2<br>Separated/divorced ……3<br>Widowed…….4		
109	Partner's occupation	Housewife……….......... 1 Farmer…..... 2<br>Merchant/ Petty trader …3 Student ....4<br>Civil servant…………… .5 Daily laborer…..6<br>Other... 88		
110	What is the highest grade of education your partner attended?	Cannot read and write ………0<br>No formal school but can read & Write. … 2<br>University/College diploma… . 3<br>Grade		
111	How many years have you lived in this location?	……………… Years		
112	Number of people lining in this household (including you)	……………………		
113	Number of under 7 years children in this household?	……………………		
114	What is your/family income (monthly)<br>'***refer last year's annual income in farmers/traders***'	………………. birr		
115	How do you perceive you're your economic states relative to other peoples in the kebele?	Poor……1 Average……2 Better of……3		
**Section 2: Information source assessment**				
201	How often do you read a newspaper or magazine?	Every day …1 Some tines …2 Not at all….3		
202	How often do you listen to the radio?	Every day …1 Some tines …2 Not at all….3		
203	How often do you watch television?	Every day …1 Some tines …2 Not at all….3		
204	Have you ever attended a health education session?	Every day …1 Some tines …2 Not at all….3		
205	How often do you participate in meetings/ conferences prepared by the kebele or the district?	Every day …1 Some tines …2 Not at all….3		
**Section 3: knowledge about Cause of lymphoedema (elephantiasis (LF or Podo or leprosy))**				
501	Lymphoedema is caused by contact with affected people	Yes …….1 No …….0		
502	Lymphoedema l is caused by bad things happening to you.	Yes …….1 No …….0		
503	Lymphoedema l is God's punishment.	Yes …….1 No …….0		
504	Lymphoedema is caused by contact with soil	Yes …….1 No …….0		

505	Lymphoedema is caused by a personal weakness	Yes …….1	No …….0		
506	Lymphoedema is randomly occurring	Yes …….1	No …….0		
507	Lymphoedema is caused by being possessed by a demon (evil spirit)	Yes …….1	No …….0		
508	Lymphoedema is caused by flies	Yes …….1	No …….0		
509	Lymphoedema is caused by mosquitos	Yes …….1	No …….0		
510	Lymphoedema is caused by stepping on snakes or dead animals	Yes …….1	No …….0		
511	Ask them if there are other causes (Please specify)	___________ _			
512	Do you know someone who has lymphoedema or foot problems? (elephantiasis: LF, podo or leprosy)	Yes …….1	No …….0		
513	Is there any one in your house who is suffering from lymphoedema?	Yes …….1	No …….0		
514	Do you have any relative or close person who has lymphoedema?	Yes …….1	No …….0		
515	What are the signs and symptoms that individuals with lymphoedemal show? ***(ask person to list)***				
**Section 4: Knowledge about people with lymphoedema due to LF, podo or leprosy**					
601	People with lymphoedema are largely to blame for their own condition.	Yes …….1	No …….0		
602	People with lymphoedema are not capable of true friendship.	Yes …….1	No …….0		
603	People with lymphoedema can work.	Yes …….1	No …….0		
604	Anyone can suffer from lymphoedema.	Yes …….1	No …….0		
**Section 5: Attitudes towards people with lymphoedema**					
701	People with lymphoedema should be prevented from having children.	Yes …….1	No …….0		
702	People with lymphoedema should not get married.	Yes …….1	No …….0		
703	People with lymphoedema should not be allowed to make decisions, even those concerning routine events.	Yes …….1	No …….0		
704	I could maintain a friendship with someone with lymphoedema.	Yes …….1	No …….0		
705	I could marry someone with lymphoedema.	Yes …….1	No …….0		
706	I would be afraid to have a conversation with someone with lymphoedema.	Yes …….1	No …….0		
707	People with lymphoedema should have the same rights as anyone else.	Yes …….1	No …….0		
708	I would be upset or disturbed about working on the same job as someone with lymphoedema.	Yes …….1	No …….0		
709	I would be ashamed if people knew that someone in my family had been diagnosed with lymphoedema.	Yes …….1	No …….0		
710	People are generally caring and sympathetic towards people with lymphoedem.	Yes …….1	No …….0		
711	Caring for people with lymphoedema is the responsibility of the family	Yes …….1	No …….0		
712	Lymphoedema is transmitted from person to person	Yes …….1	No …….0		
713	If I am served together with someone with lymphoedema, I will eat the food with no complaint	Yes …….1	No …….0		
**Section 6: Care and management of people with lymphoedema**					
801	Do you think lymphoedema is treatable?	Yes …….1	No …….0		
802	Is there a lymphoedema health service in your district?	Yes …….1	No …….0		
803	Information about lymphoedema is available at nearby health center.	Yes …….1	No …….0		

804	The best treatment for lymphoedema is medication.	Yes …….1 No …….0		
805	The best treatment for lymphoedema is holy water	Yes …….1 No …….0		
806	The best treatment for lymphoedema is with traditional healers.	Yes …….1 No …….0		
807	The best treatment for lymphoedema is prayer at Church	Yes …….1 No …….0		
808	Can you tell me any ways of treating lymphoedema?	1. Washing feet<br>2. Using soap<br>3. Using ointment<br>4. Using bandages<br>5. Using socks and shoes<br>6. Doing foot/leg exercises<br>7. Elevating the feet at night<br>8. Other (Specify) _______<br>99. I don't know		
809	As soon as a person shows signs of lymphoedema, he should be taken to holy water	Yes …….1 No …….0		
810	As soon as a person shows signs of lymphoedema, he should be taken to a witch doctor (*awaqi)*	Yes …….1 No …….0		
811	As soon as a person shows signs of lymphoedema, he should be taken to a health center	Yes …….1 No …….0		
812	People with lymphoedema have for too long been the subject of ridicule	Yes …….1 No …….0		
813	We have a responsibility to provide the best possible care for people with lymphoedema	Yes …….1 No …….0		
814	People with lymphoedema don't deserve our sympathy	Yes …….1 No …….0		
815	People with lymphoedema are a burden on society	Yes …….1 No …….0		
816	Increased spending on treatment of lymphoedema at health services is a waste of money	Yes …….1 No …….0		
817	There are sufficient existing services for people with lymphoedema	Yes …….1 No …….0		
818	People with lymphoedema shouldn't be given any responsibility	Yes …….1 No …….0		
819	Anyone with a history of lymphoedema problems should be excluded from taking public office	Yes …….1 No …….0		
820	No-one has the right to exclude people with lymphoedema from their neighborhood	Yes …….1 No …….0		

Section 7: Social Distance Scale

Please read following statement **(vignette)**

Abeba is a 27-year-old woman. She has been treated for lymphoedema during the past year. Her leg is still swollen. Abeba has a job in the local small business that belongs to her uncle. She earns Birr 10 thousands per month and is doing well in her job. She is a little bit slower than before, because of the effects of lymphoedema on her leg, but the employer never complained about that. At her job, Abeba gets along well with her colleagues. Abeba would like to get married. She is considering joining a local youth organization, so she can meet people of the same age. She also hopes to get a better job to be able to earn more than her present job.

Please, select your most fit to your opinion

901	How would you feel about renting a room in your home to someone like Abeba?	Definitely willing… 0<br>Probably not willing…2	Probably willing……1<br>Definitely not willing… 3	

902	How about being a worker on the same job with someone like Abeba?	None of time… ...... 1<br>A little of time ………2<br>Some of the time……3<br>Most of the time… ..... 4<br>All of the time… 5		
903	How would you feel having someone like Abeba as a neighbor?	None of time… ...... 1<br>A little of time ………2<br>Some of the time……3<br>Most of the time… ..... 4<br>All of the time… 5		
904	How about having someone like Abeba as caretaker of your children for a couple of hours?	None of time… ...... 1<br>A little of time ………2<br>Some of the time……3<br>Most of the time… ..... 4<br>All of the time… 5		
905	How about having one of your children marry someone like Abeba?	None of time… ...... 1<br>A little of time ………2<br>Some of the time……3<br>Most of the time… ..... 4<br>All of the time… 5		
906	How would you feel about introducing Abeba to a young woman you are friendly with?	None of time… ...... 1<br>A little of time ………2<br>Some of the time……3<br>Most of the time… ..... 4<br>All of the time… 5		
907	How would you feel about recommending someone like Abeba for a job working for a friend of yours?	None of time… ...... 1<br>A little of time ………2<br>Some of the time……3<br>Most of the time… ..... 4<br>All of the time… 5		
