# Supplementary material for: The role of a community conversation intervention in reducing stigma related to lower limb lymphoedema in Northern Ethiopia
Source: BMC Health Serv Res. 2024 Mar 19;24:353. doi: 10.1186/s12913-024-10864-w (PMC10949692; doi:10.1186/s12913-024-10864-w)
Supplement: Supplementary file 2 — Additional file 2. Patient cohort survey questionnaire. [file 12913_2024_10864_MOESM2_ESM.pdf]

Lymphoedema in Awi zone

Patient cohort Questionnaire

**INTERVIEW DETAILS**

001	Ethiopian calendar Interview date (E.C.)	**[ ] [ ] / [ ] [ ]/ [ ] [ ] [ ] [ ]**		EClaydate
002	European Calendar Interview date (G.C.)	**[ ] [ ] / [ ] [ ]/ [ ] [ ] [ ] [ ]**		GClaydate
003	Health center name			Cname
004	Health center code			cid
005	Sex (by observation)	Male	0	gend
		Female	1	

Eligibility criteria				
006	Is the respondent a resident of Guagusa Shikudad district? (Resident at least in the past 6 months)	No	0	eligres
		Yes	1	
007	Is the respondent above 18 years old?	No	0	eligage
		Yes	1	
008	Is the respondent able to hear sufficiently, i.e. the respondent does not have difficulty communicating because she/he is deaf?	No	0	eligcom
		Yes	1	
009	Is the respondent able to communicate sufficiently in Amharic?	No	0	Elignonc
		Yes	1	
010	Is the respondent healthy enough to take part, i.e. the respondent is not acutely ill or in pain?	No	0	eligill
		Yes	1	
If the is any **'0' scoring for any question between 006 and 010,** the respondent is not eligible.<br>If the **respondent is not eligible, thank him/her and discontinue**				
**If the respondent is eligible**, **proceed to consent**				

**011**	Has the respondent consented to participate in the study?	No	0	Consent
		Yes	1	
**012**	If the respondent is not willing to participate in the study, what is his/her reason?<br>Write reason briefly in the next column. (Ask in a non-coercive way about reason. If person does not want to respond, thank them and end interview)			Non consent

If the respondent is eligible and has consented to participate in the study – write their contact information below [This identifying detail will be extracted from the data and put separately for confidentiality]

013	Participant Name		PNA
014	Participant ID		PID
015	Kebele		Kebele
016	Gott		Got
017	Telephone number		Tele
018	Additional telephone number		Tele2
019	Name of the head of the household		Nhead

SECTION 1: General Information				
101	Participant's card No (complete this by looking at the participant's card)			PCNO
102	Sex (by observation)	Male	0	SEX
		Female	1	
103	Age	________________Years		age
104	Educational background (What is the highest level of education you have completed?)	Illiterate[→go to Q 106]	1	EDU
		Can read and write but didn't attend formal education (e.g learned at church or mosque or got non formal basic education) [→go to Q 106]	2	
		Attended formal education [→go to Q 105]	3	
105	If you attended formal education, up to what grade/level did you attend?<br><br>E.g Grade 6	________________		EDUYR
106	Marital status (What is your current marital status)?	Never Married	1	MARIT
		Married	2	
		Divorced	3	
		Widowed	4	
		Married but not living together	5	
		Cohabitating	6	
107	Religion (what is your religion?)	Christian	1	RELIG
		Muslim	2	
		If other [specify]____________	77	
108	Living place (where do you live, in urban or rural kebele?)	Urban	0	PLRES
		Rural	1	

109	How do you primarily spend your time in a typical day?	Paid work	1	EMP
		Private work (shopkeeper, own business, etc)	2	
		Private work (farming)	3	
		Housewife (work in the home and child care)	4	
		Study	5	
		Unemployed	6	
		Other (specify)	77	
110	When you compare yourself with other people in your neighborhood, how would you express your family's current income?	Very low	1	REINC
		Low	2	
		Middle	3	
		High	4	
		Very high	5	
111	Do you have children?	No [Skip to next section (Section 2)]	0	KIDS
		Yes	1	
112	How many children do you have?	[ ][ ]		KIDSNO
113	How old is your youngest child?	[ ][ ] year		KIDYR

SECTION 3: MMDP assessment (fill it by examining the participant)					
301	Case	LF/podo		1	CAS
		Leprosy		2	
302	Sign of bacterial/Fungal infection	Right leg	No	0	INF
			Yes	1	

		Left leg		No	0	
				Yes	1	
303	Wounds	Right leg		No	0	WOU
				Yes	1	
		Left leg		No	0	
				Yes	1	
304	Nodules	Right leg		No	0	NOD
				Yes	1	
		Left leg		No	0	
				Yes	1	
305	Swelling size	Longitudinal Circumference at the biggest swelling site measurement	Right lower leg	Long@ ______		SWS
				Size ______		
			Left lower leg	Long@ ______		
				Size ______		
		Latitudinal circumference at the biggest swelling site measurement	Right foot	Lat@ ______		
				Size ______		
			Left foot	Lat@ ______		
				Size ______		
306	Lymphoedema Stage<br><br>(Use podoconiosis lymphoedema scale)	Right leg		______		LES
		Left leg		______		
307	Leprosy disability grade					LEG
308	Has your leg ever suddenly become hot, red and painful?			No	0	AAH
				Yes	1	
309	How often does your leg become hot, red and painfull?			Every week	1	HOA
				Every two weeks	2	

	(acute attack)	Every month	3	
		Every 3 months	4	
		Every 6 months	5	
		Every year	6	
		Less often than every year	7	
310	Acute attack/reaction in the last month<br><br>(Number of episodes)			AAT
311	Have you ever recived treatment for your leg lymphoedema	No [→go to section 4]	0	LTH
		Yes [→go to Q 312]	1	
312	Are you currently receiving treatment for, or self-treating your leg(s)?	No	0	ACT
		Yes	1	
313	From where are you receiving treatment?	Government clinic	1	LTD
		Non-government clinic	2	
		Pharmacy	3	
		Traditional healer	4	
		Friend or family	5	
		Self-treatment	6	
		Other(Specify)	7	
314	Can you describe the treatment you are using?			CDT

SECTION 7: Social support scale (OSLO 3)				
701	How easy is it to get practical help from neighbors if you should need it?	Very difficult	1	OSAS
		Difficult	2	
		Possible	3	
		Easy	4	
		Very easy	5	
702	How many people are so close to you that you can count on them if you have serious personal problems (choose one option)?	None	1	OSCRS
		1 or 2	2	
		3-5	3	
		More than 5	4	
703	How much concern do people show in what you are doing (choose one option)?	Little concern and interest	1	OSNPS
		Uncertain	2	
		Some concern and interest	3	
		A lot of concern and interest	4	

**SECTION 8: Discrimination (DISC)**
In this section, I would like to ask about times in the last 6 months when you have been treated unfairly because of your lymphoedema and co-morbid mental health. In this section, there are 19 questions. Please give a response for each.

801	Have you been treated unfairly in making or keeping friends?	Not at all	0	DIFUR6
		A little	1	
		Moderately	2	
		A lot	3	
		Not applicable	99	
802	Have you been treated unfairly by the people in your neighborhoods?	Not at all	0	DNAU6
		A little	1	
		Moderately	2	
		A lot	3	
		Not applicable	99	
803	Have you been treated unfairly in dating or intimate relationships? (excluding treatment by spouse or co-habiting partner as covered by Q806)	Not at all	0	DLFUA6
		A little	1	
		Moderately	2	
		A lot	3	
		Not applicable	99	
804	Have you been treated unfairly in housing? (including becoming homeless)	Not at all	0	DHRUM6
		A little	1	
		Moderately	2	
		A lot	3	
		Not applicable	99	
805	Have you been treated unfairly in your education? (ask about school, college, university, on the job training, vocational courses)	Not at all	0	DECUT6
		A little	1	
		Moderately	2	
		A lot	3	

		Not applicable	99	
806	Have you been treated unfairly in marriage or divorce? (including co-habiting or civil partnership. Ask about ability to find a partner or spouse, problems during the relationship, divorce settlements)	Not at all	0	DMRD6
		A little	1	
		Moderately	2	
		A lot	3	
		Not applicable	99	
807	Have you been treated unfairly by your family? (ask about family of origin – parents, brothers, sisters and other relations as well as any children. Exclude treatment by spouse or co-habiting partner as covered by Q806)	Not at all	0	DFBSR6
		A little	1	
		Moderately	2	
		A lot	3	
		Not applicable	99	
808	Have you been treated unfairly in finding a job? (this means finding full or part-time paid work)	Not at all	0	DGWU6
		A little	1	
		Moderately	2	
		A lot	3	
		Not applicable	99	
809	Have you been treated unfairly in keeping a job?	Not at all	0	DWEU6
		A little	1	
		Moderately	2	
		A lot	3	
		Not applicable	99	
810	Have you been treated unfairly when using public transport? (ask about using free travel pass, passengers, drivers, etc)	Not at all	0	DPRTU6
		A little	1	
		Moderately	2	

		A lot	3	
		Not applicable	99	
811	Have you been treated unfairly in your religious practices? (ask about attending church, other church members, church leaders)	Not at all	0	DUDBO6
		A little	1	
		Moderately	2	
		A lot	3	
		Not applicable	99	
812	Have you been treated unfairly in your social life? (ask about socializing, hobbies, attending events, leisure activities)	Not at all	0	DSLPT6
		A little	1	
		Moderately	2	
		A lot	3	
		Not applicable	99	
813	Have you been treated unfairly when getting help for physical health problems? (ask about GP, dentist, nurses, health officers, health extension workers, and emergency treatment)	Not at all	0	DPBHP6
		A little	1	
		Moderately	2	
		A lot	3	
		Not applicable	99	
814	Have you been treated unfairly in your personal safety and security? (ask about verbal abuse, physical abuse, assault)	Not at all	0	DPSRT6
		A little	1	
		Moderately	2	
		A lot	3	
		Not applicable	99	
815		Not at all	0	DFCPD6

	Have you been treated unfairly in starting a family or having children? (ask about the behavior of health professionals, friends and family, as well as how they or their partner were treated during pregnancy or childbirth)	A little	1	
		Moderately	2	
		A lot	3	
		Not applicable	99	
816	Have you been treated unfairly in your role as a parent to your children? (ask about behavior of other parents, teachers, family or health staff)	Not at all	0	DFRT6
		A little	1	
		Moderately	2	
		A lot	3	
		Not applicable	99	
817	Have you been treated unfairly in your levels of privacy? (ask about privacy in hospital and in community settings, eg private letters or phone calls, medical records)	Not at all	0	dpriv6
		A little	1	
		Moderately	2	
		A lot	3	
		Not applicable	99	
818	How much do you agree with the following statement:<br>I feel that receiving treatment for my lymphoedema and co-morbid mental health problems has led to people tending to treat me more fairly	Strongly agree	1	dtreat6
		Agree	2	
		Neither agree / disagree	3	
		Disagree	4	
		Strongly disagree	5	
819	*Main source of information for DISC*	Patient	1	discinfo6
		Caregiver	2	
		Both	3	

SECTION 9: Internalized Stigma Related to Lymphoedema (ISRL)				
We are going to use the term "lymphoedema illness" in the rest of this questionnaire, but please think of it as whatever you feel is the best term for it.<br><br>I will ask you some questions about these problems. Let me know if you agree or disagree with the following statements.				
901	I feel out of place in the world because of my illness	Strongly disagree	1	_ISMI01
		Disagree	2	
		Agree	3	
		Strongly agree	4	
902	I am embarrassed or ashamed of these problems	Strongly disagree	1	_ISMI05
		Disagree	2	
		Agree	3	
		Strongly agree	4	
903	I am disappointed in myself due to these problems	Strongly disagree	1	_ISMI16
		Disagree	2	
		Agree	3	
		Strongly agree	4	
904	These problems have spoiled my life	Strongly disagree	1	_ISMI17
		Disagree	2	
		Agree	3	
		Strongly agree	4	
905	Because of these problems, I need others to make most decisions for me	Strongly disagree	1	_ISMI19
		Disagree	2	
		Agree	3	
		Strongly agree	4	

906	I can't contribute anything to society because of these problems	Strongly disagree	1	_ISMI23
		Disagree	2	
		Agree	3	
		Strongly agree	4	
907	People discriminate against me due to these problems	Strongly disagree	1	_ISMI03
		Disagree	2	
		Agree	3	
		Strongly agree	4	
908	People often patronize me, or treat me like a child, just because of these problems	Strongly disagree	1	_ISMI15
		Disagree	2	
		Agree	3	
		Strongly agree	4	
909	People ignore me or take me less seriously just because of these problems	Strongly disagree	1	_ISMI22
		Disagree	2	
		Agree	3	
		Strongly agree	4	
910	Nobody would be interested in getting close to me because of these problems	Strongly disagree	1	_ISMI25
		Disagree	2	
		Agree	3	
		Strongly agree	4	
911	Others think that I can't achieve much in life because of these problems	Strongly disagree	1	_ISMI28
		Disagree	2	
		Agree	3	
		Strongly agree	4	
